# Palatoplasty in children: nursing diagnoses and interventions related to the immediate postoperative period

**DOI:** 10.1590/1980-220X-REEUSP-2020-0252

**Published:** 2022-01-05

**Authors:** Ana Flávia Martinez, Nayara Tomazi Batista, Gesiane Cristina Bom, Cláudia Regina Matiole, Carmen Silvia Zamboni, Armando dos Santos Trettene

**Affiliations:** 1 Universidade de São Paulo, Hospital de Reabilitação de Anomalias Craniofaciais, Programa de Residência Multiprofissional em Síndromes e Anomalias Craniofaciais, Departamento de Enfermagem, Bauru, SP, Brazil.; 2 Universidade de São Paulo, Hospital de Reabilitação de Anomalias Craniofaciais, Departamento de Enfermagem, Bauru, SP, Brazil.

**Keywords:** Nursing Process, Cleft Palate, Nursing Diagnosis, Nursing Care, Postoperative Period, Standardized Nursing Terminology., Procesos de Enfermería, Fisura del Paladar, Diagnóstico de Enfermería, Cuidados de Enfermagem, Periodo Posoperatorio, Terminología Normalizada de Enfermería, Processos de Enfermagem, Fissura Palatina, Diagnóstico de Enfermagem, Cuidados de Enfermagem, Período Pós-Operatório, Terminologia Padronizada em Enfermagem

## Abstract

**Objective:**

To identify the main nursing diagnoses and interventions in children in the immediate postoperative period of palatoplasty.

**Method:**

Documentary and retrospective study, developed in a Brazilian public and tertiary hospital, between August and September 2020. Children who underwent only palatoplasty, between January and December 2019, aged between 10 and 24 months, were included. Those with medical syndromes and/or comorbidities were excluded. The Theoretical Framework of Basic Human Needs and the NANDA International and Nursing Interventions Classification Taxonomies were used. Data underwent descriptive statistical analysis.

**Results:**

The sample consisted of 126 children. Psychobiological needs such as oxygenation, hydration, nutrition, elimination, cutaneous-mucosal and physical integrity, pain and environmental perception predominated. Based on them, nine nursing diagnoses, with four focusing on the problem and five on risk, as well as 17 interventions, were identified.

**Conclusion:**

The use of standardized languages to identify affected human needs and, based on them, diagnoses and interventions, favored clinical reasoning for the construction and organization of clinical nursing practice.

## RESUMO

**Objetivo:**

identificar os principais diagnósticos e intervenções de enfermagem em crianças em pós-operatório imediato de palatoplastia.

**Método:**

estudo documental e retrospectivo, desenvolvido em um hospital público e terciário brasileiro, entre agosto e setembro de 2020. Foram incluídas crianças submetidas exclusivamente a palatoplastia, que realizaram a cirurgia entre janeiro e dezembro de 2019, com idade entre 10 e 24 meses. Excluíram-se aquelas com síndromes e/ou comorbidades clínicas. Utilizou-se o Referencial Teórico das Necessidades Humanas Básicas e as Taxonomias NANDA Internacional e da *Nursing Interventions Classification*. Os dados foram submetidos a análise estatística descritiva.

**Resultados:**

a amostra constou de 126 crianças. Predominaram as necessidades psicobiológicas: oxigenação, hidratação, nutrição, eliminação, integridade cutâneo-mucosa e física, percepção dolorosa e ambiental. A partir delas foram identificados 9 diagnósticos de enfermagem, sendo 4 com foco no problema e 5 de risco, além de 17 intervenções.

**Conclusão:**

a utilização de linguagens padronizadas na identificação das necessidades humanas afetadas e, a partir delas os diagnósticos e intervenções, favoreceu o raciocínio clínico para a construção e organização da prática clínica de enfermagem.

## RESUMEN

**Objetivo:**

identificar los principales diagnósticos e intervenciones de enfermería en niños en postoperatorio inmediato de palatoplastía.

**Método:**

estudio documental y retrospectivo, desarrollado en un hospital público y terciario brasileño, entre agosto y septiembre de 2020. Se incluyeron niños sometidos exclusivamente a palatoplastía, que realizaron la cirugía entre enero y diciembre de 2019, con edad entre 10 y 24 meses. Se excluyeron aquellos con síndromes y/o comorbidades clínicas. Se utilizó el Referencial Teórico de las Necesidades Humanas Básicas y las Taxonomías NANDA Internacional y la *Nursing Interventions Classification*. Los datos fueron sometidos al análisis estadístico descriptivo.

**Resultados:**

la muestra constó de 126 niños. Predominaron las necesidades psicobiológicas: oxigenación, hidratación, nutrición, eliminación, integridad cutáneo-mucosa y física, percepción dolorosa y ambiental. A partir de eso se identificaron 9 diagnósticos de enfermería, siendo 4 con enfoque en el problema y 5 de riesgo, además de 17 intervenciones.

**Conclusión:**

la utilización de lenguajes estandarizado en la identificación de las necesidades humanas afectadas y, a partir de ellas los diagnósticos e intervenciones, favoreció el raciocinio clínico para que se construyese y se organizase la práctica clínica de enfermería.

## INTRODUCTION

Cleft lip and/or palate are prevalent among the malformations affecting the face, being defined as openings or interruptions in the lip and/or oral cavity structures, the location and extension of which are variable. In Brazil, the incidence is 1:650 live births. The etiology involves both genetic and environmental factors, that is, it is multifactorial^(1,2)^.

They result from failures in the fusion of facial processes that take place between the 4th and 12th gestational week and can be classified according to the anatomical location in cleft lip, palate, and lip and palate. As for the extension, they can be complete or incomplete, uni- or bilateral^(1–3)^.

Patients with this malformation may present aesthetic, functional, and psychosocial problems. When the palate is affected, the surgery is called palatoplasty, which can be performed using different surgical techniques, depending on the classification of the cleft, anatomical amplitude, repair time (1 or 2 times), experience of the surgeon, and child’s general health status^(1,4)^.

Although there is no consensus regarding the age and ideal surgical technique, in the hospital that is the setting for this research, palatoplasty is performed from one year onwards and its benefits are particularly related to voice quality^(1,5,6)^.

Among the surgery complications, those related to the respiratory system are highlighted, such as airway obstruction, foreign body aspiration, laryngeal spasm, oxygen desaturation, and need for re-intubation. Others, less frequent, include hemorrhage or bleeding, nausea and vomiting^(7,8)^. They occur predominantly in the immediate postoperative period (IPO), that is, in the first 24 hours after the surgical procedure, and require special attention from nursing professionals^(9,10)^.

In this regard, nursing professionals use the Nursing Care Systematization as a methodology to plan, organize, and direct their practice, where the operationalization takes place through the elaboration of the Nursing Process (NP). This, as far as it is concerned, consists of the following steps: history (interview and physical examination), diagnosis, prescription or interventions, and nursing assessment or progress evaluation, as recommended by Resolution 358/2009 of the Federal Nursing Council, which should be based on a Theoretical Framework^(11,12)^.

Despite the differences among researchers regarding the classification in theoretical or conceptual framework, the one of Basic Human Needs proposed by Wanda Aguiar Horta is in line with the mission/philosophy of the institution that is the setting of this research, and is the most used in Brazil to promote the NP stages. Its presuppositions claim that needs are universal, although the way of manifestation and satisfaction varies from one individual to another. It is based on general laws that govern universal phenomena of dynamic balance, adaptation and holism, and interrelates the concepts of human being, environment and nursing, where human needs are organized into three dimensions: psychobiological, psychosocial, and psychospiritual^(13)^.

Aiming to systematize and universalize the NP, and make it more uniform and representative, standardized languages or taxonomies were developed, including the NANDA International (NANDA-I) classification, Nursing Interventions Classification (NIC) and the Nursing Outcomes Classification (NOC), which refer, respectively, to nursing diagnoses, interventions, and outcomes^(14–16)^.

For the present study, nursing diagnoses and interventions were considered. The former consists of “clinical judgment related to a poor response or about the individual’s vulnerability to a health condition or for the maintenance and improvement of well-being of an individual, family, or community”^(14)^. Nursing interventions, as far as they are concerned, refer to actions that consider nurses’ clinical judgment and knowledge, with an emphasis on the search for and improvement of results, which may be individual or collective, with direct or indirect care^(15)^.

Based on the above, we sought to answer the following question: what are the nursing diagnoses and interventions in the IPO of children undergoing palatoplasty? Although studies on nursing diagnoses and interventions in the pediatric context are available, those aimed at children with cleft lip and/or palate are incipient, including those related to the postoperative situation.

Furthermore, the identification of nursing diagnoses and interventions in clients with specific health conditions contributes to consolidating the body of knowledge based on scientific evidence, allowing knowledge of the main care needs, thus allowing the planning of more assertive, safe, and quality nursing activities, as well as providing the construction of protocols and recording instruments. Moreover, these factors in association favor professional recognition by providing visibility to the profession, as they allow the formalization of a specific body of knowledge.

This way, we aimed at identifying the main nursing diagnoses and interventions in children in palatoplasty IPO.

## METHOD

### Design of Study

This is a documentary and retrospective study with a quantitative design.

### Local

The study was carried out in a public tertiary hospital located in an inland city of the state of São Paulo, Brazil. It is a national and international reference institution in the care of patients with craniofacial anomalies and associated syndromes. It is managed by Universidade de São Paulo, with resources from the Brazilian Public Health System, and operates in the healthcare, teaching, and research areas. It provides multidisciplinary and interdisciplinary assistance.

### Sample Selection and Definition Criteria

Children who underwent solely palatoplasty, who were in IPO, that is, within the first 24 hours after the procedure, who underwent surgery between January and December 2019, aged between 10 and 24 months, were included. Syndromic children and/or those with medical comorbidities, such as cardiopathies and pneumopathies, were excluded.

Initially, 230 children were selected. Of these, 53 underwent other concomitant surgeries, 38 had associated syndromes, and 13 had clinical comorbidities. Finally, the sample consisted of 126 children.

### Data Collection

Data collection took place between August and September 2020. For this, initially, data from the nursing history were considered, which included anamnesis and physical examination. Subsequently, the diagnoses and nursing interventions listed in the medical record were considered.

Although the electronic medical record is under implementation process in the institution, which is the setting for this research, it is still formalized manually through an instrument that was built and validated for this purpose, prior to this study, based on the Framework of Basic Human Needs by Wanda de Aguiar Horta, NANDA-I and NIC Taxonomies^(13–15)^. The latest version of that instrument was implemented in 2018 and all nurses at the institution were trained to use it. Among them, there are researchers in this field, with study groups and publications. It describes the main nursing diagnoses and interventions, listed in a checklist. In addition to these, there is space to include other diagnoses, as well as other interventions not covered in the form.

Aiming to make the findings more reliable, besides eliminating possible biases, we sought to assess the percentage of agreement among the findings described in the medical record and those evidenced by the researchers regarding nursing diagnoses and interventions. Thus, for data collection, an instrument designed and validated for the present study was used, based on the authors’ experience, where pertinent information was thoroughly described, that is, all possible nursing diagnoses and interventions were listed. Two nurses, authors of this investigation, collected data independently. In cases of divergence, the advisor researcher was consulted. Both professionals were trained/calibrated through a pre-test, which included 10 participants.

In addition, the participants were characterized according to the variables: age, sex, socioeconomic classification, average length of stay in the hospital, and cleft classification. For the socioeconomic classification, the one used as a protocol in the institution, field of this research, was considered, the framework of which addresses the following indicators: family economic situation, family composition, level of education and occupation, housing condition and situation, among others, systematized in an instrumental with a scoring system that allows the classification of family reality into one of six stratifications, namely: lower low, higher low, lower average, average, higher average, and high^(17)^.

### Data Analysis and Treatment

The NP construction was based on the Framework of Basic Human Needs by Wanda de Aguiar Horta. Considering that the target audience consisted of children, the psychobiological and psychosocial dimensions were particularly considered^(13)^.

Nursing diagnoses were classified according to NANDA-I, as for their typology, that is, focusing on the problem, syndromes, health promotion, and risk, while interventions were listed according to the NIC^(14,15)^. Data were tabulated using the software Excel 2016^®^ and the results were subjected to descriptive statistical analysis.

### Ethical Aspects

Data collection began after approval by the Human Research Ethics Committee at the Hospital, as per opinion 3.781.317 of December 2019. As this is a study using a secondary source of data, that is, where patient records were retrospectively analyzed, a Term of Commitment by the Researchers was formalized regarding the use and anonymity of the information obtained, in accordance with the precepts of Resolution 466/2012 of the National Health Council.

## RESULTS

The sample consisted of 126 children, whose mean age was 14 months (SD = 2.55). Regarding sex, 52% (n = 65) were women. As for social classification, 41% (n = 52) belonged to the higher low. Regarding the cleft category, the lip and palate prevailed. It should be noted that, in this category of cleft, lip surgery, cheiloplasty, is performed at 3 months, while palatoplasty, palate surgery, is performed after 1 year.

The basic human needs affected consisted of psychobiological (oxygenation, hydration, nutrition, elimination, cutaneous-mucosal and physical integrity, pain and environmental perception) and psychosocial (safety) dimensions.

Four nursing diagnoses focused on the problem and five on risk were identified. Among those focusing on the problem, 83% (n = 105) listed the integrity of the impaired oral mucous membrane, related to the surgical procedure, characterized by oral discomfort (Table 1).

**Table 1.** Distribution of nursing diagnoses focusing on the problem in children in the immediate postoperative period of palatoplasty. Bauru, SP, Brazil, 2020.

Nursing diagnoses	n	%
INTEGRITY OF THE IMPAIRED ORAL MUCOUS MEMBRANE (00045)	105	83
**Defining characteristics**		
Oral discomfort	105	83
Oral edema	57	45
**Related factor**		
Surgical procedure	105	83
**Related factor**		
Edema (surgical procedure)	84	67
ACUTE PAIN (00132)	88	70
**Defining characteristics**		
Change in appetite	64	51
Expressive behavior	24	19
**Related factor**		
Harmful physical agent	88	70
INEFFECTIVE RESPIRATORY PATTERN (00032)	84	67
**Defining characteristics**		
Nasal flaring	74	59
Abnormal breathing pattern	60	48
Breath with pursed lips	36	29
Use of accessory muscles to breathe	84	67
**Related factor**		
Edema (surgical procedure)	84	67
INEFFECTIVE INFANT FEEDING PATTERN (00107)	78	62
**Defining characteristics**		
Inability to initiate effective suction	78	62
**Associated condition**		
Oral hypersensitivity	78	62

**Chart 1.**
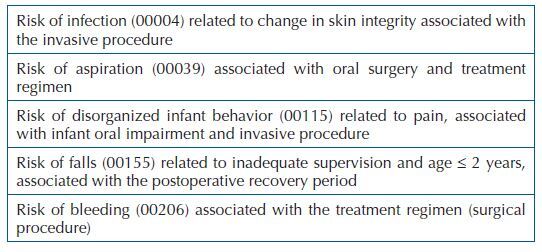
Risk nursing diagnoses with their respective related factors and/or associated conditions, in children in the immediate postoperative period of palatoplasty. Bauru, SP, Brazil, 2020.

As for risk nursing diagnoses, 100% (n = 126) showed risk of infection, risk of aspiration, risk of disorganized infant behavior, risk of falls, and risk of bleeding, whose related factors and/or associated conditions are described in Chart 1.

Regarding nursing interventions, among the 17 listed, it was observed, in 100% (n = 162) of them: airway aspiration, care with the incision site, pain control, analgesics administration, calming techniques, vital signs monitoring, infection protection, aspiration precautions, post-anesthesia care, anxiety reduction, infant care, prevention of falls, environmental control: safety, and precautions against bleeding (Table 2).

**Table 2.** Distribution of nursing interventions listed for children in the immediate postoperative period of palatoplasty. Bauru, SP, Brazil, 2020.

Nursing interventions	n	%
Airway aspiration (3160)	126	100
Incision site care (3440)	126	100
Pain control (1400)	126	100
Analgesics administration (2210)	126	100
Calming technique (5880)	126	100
Vital signs monitoring (6680)	126	100
Infection protection (6550)	126	100
Precautions against aspiration (3200)	126	100
Post-anesthesia care (2870)	126	100
Anxiety reduction (5820)	126	100
Baby care (6820)	126	100
Prevention of falls (6490)	126	100
Environment control: safety (6486)	126	100
Precautions against bleeding (4010)	126	100
Restoration of oral health (1730)	105	83
Airway control (3140)	84	67
Nutrition control (1100)	78	62

## DISCUSSION

Children’s mean age was 14 months. Although it is not a consensus, according to the protocol of the institution that is the setting for this research, palatoplasty shall be performed after 12 months. It should be noted that compliance with the therapeutic protocol is essential in the rehabilitation process, aiming at the best results, which in palatoplasty refers mainly to voice quality^(1)^.

Regarding sex, the female prevailed, corroborating the literature that indicates the prevalence of clefts with involvement of the palate among women^(1)^. As for the socioeconomic classification, higher low prevailed, showing that, in general, families consisted of four people, on average, with an income of 2 to 4 minimum wages, with education level up to some elementary school, with their own, but unsatisfactory, residence, and formed by salaried workers^(17)^.

This datum reveals the profile of patients cared for at the institution, most of whom belong to the less favored social classes, requiring, among others, special attention from the nursing team regarding postoperative instructions that shall be objective and simplified as much as possible. In addition, it is necessary to provide the community with resources for the maintenance of care after hospital discharge, to minimize complications^(17–19)^.

As for the cleft classification, the lip and palate classification prevailed, in accordance with the literature. The more anatomically complex the malformation, the more interventions will be needed. In this case, in addition to cheiloplasty (lip correction), which is performed at three months, palatoplasty is required, that is, the correction will take place at least in two stages^(1,6)^.

In this study, the basic human needs affected consisted of psychobiological (oxygenation, hydration, nutrition, elimination, cutaneous-mucosal and physical integrity, pain and environmental perception) and psychosocial (safety) dimensions. This clinical reasoning allows for the individualized assessment of the patient’s needs, as well as of the problems regarding nursing, planning, and implementation of care, as well as for the evaluation of results^(12,13)^.

Regarding the problem-focused nursing diagnoses, the Integrity of the impaired oral mucous membrane prevailed (00045), and is defined as an injury to the lips, soft tissues, oral cavity and/or oropharynx, being related to the surgical procedure and characterized by discomfort and edema^(14)^.

Considering that the cleft, as well as the surgical procedure, are located in the oral cavity, injuries and edema are frequent, mainly related to surgical trauma, besides discomfort related to the intubation procedure and the use of the orotracheal tube. Furthermore, in cases where the cleft palate is very wide, it is necessary to use releasing incisions, also called relaxers, which will make muscle mobility possible by reducing tension, minimizing the occurrence of fistulas, and favoring suture performance^(1,6,10)^. In this regard, the main interventions were related to the Restoration of oral health (1730) and Care with the incision site (3440), through monitoring of lesions and edema, pharmacological measures, and pain control^(10,15)^.

The ineffective breathing pattern (00032) is defined as inspiration and/or expiration that does not provide adequate ventilation, and was related to the surgical procedure in the oral cavity with consequent edema of the palate and tongue, which in extreme situations can cause airway obstruction. It was characterized by nasal flaring, abnormal breathing pattern, pursed-lip breathing, and use of accessory muscles^(14)^.

In fact, children undergoing palatoplasty may present transient respiratory obstruction due to the reconstruction/manipulation of anatomical structures in the oral and nasopharyngeal region, which promote changes in airflow dynamics^(20)^. The main interventions were Airway Aspiration (3160), Airway Control (3140), and Vital Signs Monitoring (6680), whose activities included monitoring of breathing pattern, oxygen saturation, peripheral perfusion, maintenance of airway permeability, administration of properly humidified oxygen therapy, among others^(15)^.

Regarding Impaired Comfort (00214), it is defined as the perception of lack of comfort, relief and transcendence in the physical, psycho-spiritual, environmental, cultural and/or social dimensions. In this study, it was related to the treatment regimen (surgery) and was characterized by the level of crying, irritability, and restlessness^(14)^. Another investigation that included children in a postoperative situation showed similar results^(21)^.

It is inferred that this nursing diagnosis is related to the change of environment, that is, from the home to the hospital, and to the performance of invasive procedures that generate insecurity and discomfort, hunger, inability to use utensils such as baby bottles, and pain. In this regard, the implementation of some interventions is critical, such as Calming Techniques (5880), Reduction of anxiety (5820), and Caring for babies (6820), including, among others, noise and light control, pain monitoring, administration of analgesics, provision of food as soon as possible, and recreational activities^(15,21)^.

In addition to the high load of physical and sensory stimulation the children receive during hospitalization, it should be considered that changing the environment and the routine intensifies the perception of discomfort^(22,23)^. Besides this, there is the immobilization of the humerus-radial joint with the use of bracelets, which aims to prevent the child from placing the hand in the oral cavity and/or perioral region, minimizing the risk of infection and injury to the surgical wound that, although necessary, causes discomfort. Also, in addition to the care previously reported, upper limb massage is necessary to promote comfort^(10)^.

Although less frequent, another nursing diagnosis listed was Acute Pain (00132), which refers to an unpleasant sensory or emotional experience associated with an injury, which has a sudden or slow onset, with mild to severe intensity and duration of less than 3 months. It was related to a harmful physical agent (surgery) and was characterized by changes in appetite and expressive behavior^(14)^. It should be noted that, in children, non-verbal language is the most used method to identify and monitor pain, as well as the perception of parents, who are familiarized with their children^(21)^.

The institutional protocol of analgesia, which included the administration of medication at regular intervals, certainly contributed for this diagnosis not to prevail. Among the interventions, the administration of analgesics (2210) and Pain control (1400) through pharmacological and non-pharmacological methods were highlighted, as well as monitoring through verbal and non-verbal indicators^(15,24)^.

The ineffective standard diagnosis of infant feeding (00107) was listed, being common in surgeries performed in the oral cavity, such as palatoplasty, related to the presence of edema, pain, and surgical manipulation. The associated condition was oral hypersensitivity^(14)^. In fact, feeding these children is sometimes a challenge, especially in the IPO. Associated with this, insufficient food acceptance, if persistent, causes nutritional imbalance, which, as far as it is concerned, may have negative repercussions on postoperative recovery^(25)^.

In addition to the factors mentioned above, the impossibility of using utensils such as baby bottles and pacifiers, which until then were common, increased the difficulty of accepting food. In this sense, to minimize this problem, the use of these utensils shall be discouraged before the surgical procedure, being replaced by a cup or spoon^(10,26)^. Moreover, it is essential to establish analgesia protocols, aiming to minimize pain. In general, the use of traditional pain relievers such as dipyrone is sufficient. However, in some cases, the association of anti-inflammatory drugs and even opioids^(10,21)^ is required.

In summary, the main intervention referred to Nutrition Control (1100), as well as to nursing activities that include: administering medication as needed, monitoring/observing the face of pain, providing family support, monitoring food acceptance, offering pasty and/or liquid food, at cold or ambient temperature, for a period of 30 days^(10,15,25)^.

Risk diagnoses were prevalent among those listed in this study, being used to reduce the severity or occurrence of that risk in the patient^(14)^. Among them, the Risk of Infection (00004) stood out, as the prevention of infection is essential for the surgical safety and patients’ health; in addition, oral cavity surgeries are considered potentially contaminated because the mucosa or tissue is colonized by microorganisms^(27)^.

The risk factors/associated conditions referred to changes in skin integrity and invasive procedures^14^. The main intervention is Protection against Infection (6550), whose activities included the promotion of oral hygiene after meals and/or when necessary, monitoring of the integrity of the oral cavity, aspects of the surgical incision, dressings, halitosis, and the presence of phlogistic signs^(15,28)^.

Another diagnosis identified was Risk of aspiration (00039), defined as susceptibility to the entry of oropharyngeal secretions, solids or liquids into the tracheobronchial passages, which could compromise health^(14)^. The main interventions are related to Precautions against aspiration (3200) and Post-anesthesia care (2870), whose activities included monitoring the breathing pattern, oxygen saturation, aspiration of secretions, and keeping the head of the bed elevated from 30° to 45°^(15)^.

Being hospitalized, even for a short period, associated with a low understanding of what is happening, in addition to the manipulation of anatomical structures, makes the child run the risk of presenting non-standard behavior, leading to the diagnosis of Risk for disorganized infant behavior (00115), which refers to the susceptibility in the modulation of physiological and neurobehavioral systems that can compromise health. In this study, related factors included pain and food intolerance, which are associated with an infant’s oral impairment and the invasive procedure^(14)^. The main interventions were focused on Pain Control (1400), Oral Health Restoration (1730), Nutrition Control (1100) and Calming Techniques (5880), besides providing family support and promoting adequate sleep^(15,23,24)^.

Another risk that is present in the hospital environment, which can be applied to any patient undergoing a procedure that requires the use of sedation or local and/or general anesthetics, includes the Risk of falls (00155), which refers to increased susceptibility to falls that can lead to physical harm and compromise health. For participants in this study, risk factors included inadequate supervision, being 2 years old or less, and the postoperative recovery period^(14)^. Others include the fact of being in an adverse moment, the hospitalization, and the change in the environment^(21)^.

In fact, the risk of falls is common among pediatric patients. Interventions can focus on both the patient and the environment, including Prevention of Falls (6490) and Environment Control: Safety (6486), through activities such as keeping crib rails raised and wheels locked, implementing scales that point out the environmental risk, emphasizing to the companion that he/she has always to warn when leaving the bed and monitor the level of consciousness and psychomotor agitation^(15,21,29,30)^.

Another diagnosis listed was the Risk of Bleeding (00206), defined as the susceptibility to a reduction in the volume of blood that can compromise health, with the associated condition of the treatment regimen, surgical procedure^(14)^. Indeed, the palatal region is highly vascularized, which predisposes to the risk of bleeding^(9–11)^.

Therefore, some interventions are needed, including Precautions against bleeding (4010)^(15)^. In addition to these, other specific ones should be used, such as using bracelets that help prevent the child from putting their hands to their mouths, avoiding the use of sharp toys, avoiding agitation and intense exposure to the sun. At the same time, the diet should be liquid and cold^(10,21)^. Moreover, cryotherapy should be performed when prescribed, and the integrity of the oral cavity, aspects of the surgical incision, dressings, and bleeding^(10,15)^ shall be monitored.

Finally, although the resolution of possible biases is the main focus, the fact that this study considers information from secondary sources may somehow influence the results, being considered a limitation.

However, the contributions of this investigation are evident, given that, in accordance with the principles of the Brazilian Public Health System, with regard to the decentralization of services, children with cleft lip and/or palate are being cared for in different health units. Thus, the knowledge established here may support the care of children undergoing palatoplasty regarding IPO, a period considered the most critical one.

Furthermore, the study has an innovative character by promoting the generation of possible health indicators, and favors nurses’ clinical reasoning in the construction of their own body of knowledge through the adoption of terminologies that contribute to nursing care.

## CONCLUSION

The use of standardized languages to list diagnoses and interventions favored clinical reasoning for the construction and organization of clinical nursing practice, also contributing to a body of specific knowledge aimed at children in postoperative palatoplasty situations.

Briefly, the findings allowed drawing a care profile aimed at the real needs of the clientele, contributing to comprehensive, humanized, safe and quality care. Considering that the knowledge produced from studies on nursing diagnoses and interventions provide a scientific basis for care planning, further investigations are required to consolidate and expand knowledge.
